# Toward a new model of human resource management practices: construction and validation of the High Wellbeing and Performance Work System Scale

**DOI:** 10.3389/fpsyg.2023.1151781

**Published:** 2023-05-10

**Authors:** Annick Parent-Lamarche, Julie Dextras-Gauthier, Anne-Sophie Julien

**Affiliations:** ^1^Département de gestion des ressources humaines, Université du Québec à Trois-Rivières, Trois-Rivières, QC, Canada; ^2^Département de Management, Université Laval, Québec, QC, Canada; ^3^Département de mathématiques et de statistique, Université Laval, Québec, QC, Canada

**Keywords:** human resource management practices, wellbeing, job performance, scale construction and validation, factorial analysis, high wellbeing and performance work system scale

## Abstract

**Introduction:**

The integrated mutual gains model suggests five provisional sets of human resource management (HRM) practices that should benefit both employees and organizations and, as such, be explicitly designed to have a positive impact on wellbeing, which, in turn, can affect performance.

**Methods:**

An extensive review of the literature on scales that used a high-performance work system to assess HRM practices, as well as an extraction of items related to the theoretical dimensions of the integrated mutual gains model, were performed. Based on these preliminary steps, an initial scale with the 66 items found most relevant in the literature was developed and assessed regarding its factorial structure, internal consistency, and reliability over a two-week period.

**Results:**

Exploratory factorial analysis following test -retest resulted in a 42-item scale for measuring 11 HRM practices. Confirmatory factor analyses resulted in a 36-item instrument for measuring 10 HRM practices and showed adequate validity and reliability.

**Discussion:**

Even though the five provisional sets of practices were not validated, the practices that emerged from them were assembled into alternative sets of practices. These sets of practices reflect HRM activities that are considered conducive to employees’ wellbeing and, consequently, their job performance. Consequently, the “High Wellbeing and Performance Work System Scale” was created. Nonetheless, future research is necessary to evaluate the predictive capacity of this new scale.

## Introduction

The relationship between human resource management (HRM) practices, wellbeing, and performance has been conceptualized in several different ways in the extant literature (Peccei and Van De Voorde, 2019). Models, namely mutual gains, conflicting outcomes, or mutual losses, can be distinguished according to the nature of the mediation (wellbeing as the mediator) involved between HRM and job performance (Peccei and Van De Voorde, 2019). From a mutual gains’ perspective (win–win situation), HRM is supposed to have a positive effect on job performance via employees’ wellbeing. In contrast, according to the conflicting outcomes’ perspective (win–lose situation, also called zero-sum game or trade-off), HRM is assumed to lower employees’ wellbeing in order to increase their job performance, therefore implying that job performance is achieved only at the expense of employees’ wellbeing. According to Peccei (2004), HRM leads to greater intensification and monitoring of work and to a generally more systematic exploitation of employees, which could be harmful to their wellbeing. For its part, the mutual losses’ perspective also presumed that HRM is negatively associated with employees’ wellbeing, but this negative relationship presumably decreases job performance. What currently draws researchers’ attention to these relationships is the lack of detailed theoretical elaboration regarding the key links between HRM, wellbeing, and job performance (Peccei and Van De Voorde, 2019). More precisely, the link between HRM and wellbeing is poorly understood (Peccei and Van De Voorde, 2019). According to these authors, these gaps require more systematic attention to strengthen the theoretical underpinnings of HRM, wellbeing, and job performance.

Moreover, Peccei and Van De Voorde (2019) highlighted the lack of consensus and clarity surrounding the conceptualization and measurement of HRM. Beijer et al. (2021) also concluded in their critical review of HRM measurements that there was a lack of clarity and transparency in the construction of measurement scales, a clear rationale behind the choice of items. According to these authors, the most problematic issues are related to the failure in many cases to fully report: (1) the specific items used to measure perceived HRM practices, (2) the rationale behind the choice of items, and (3) the basic psychometric properties of the scales involved. In sum, what emerges from recent literature in the field is the need to push the reflections further in order to fill certain clearly identified gaps regarding the links between HRM, wellbeing, and job performance, as well as the way to measure HRM practices adequately.

### Aim of the study

We propose to elaborate on a new HRM practices scale with a clear and transparent theoretical rationale for the choice of items. These choices are based on the integrated mutual gains model proposed by Guest (2017), which is in line with the mutual gains’ perspective presented above. This model focuses on the link between HRM and employees’ wellbeing, which was identified as poorly understood by Peccei and Van De Voorde (2019). Additionally, we believe that from an ethical point of view, job performance should not be achieved at the expense of employees’ wellbeing but should rather be a positive consequence of it. Accordingly, the main objective of this study is to elaborate and validate a more complete scale for measuring HRM practices based on existing scales [high-performance work system (HPWS)], but elements related to wellbeing are prioritized; therefore, a new scale called the “High Wellbeing and Performance Work System” (HWBPWS) is created. The intention is to move away from the dominant measurement scales focused on performance (HPWS and conflicting outcomes perspective) by prioritizing the appropriate HRM practices that are assumed to prioritize employees’ wellbeing. Notably, the validation of this measurement scale in the French version can initiate, as a future research avenue, the validation of an English version based on English-speaking respondents.

### Context

In Canada, labor shortages have become one of the main concerns of organizations, as, recently, employers are having difficulties filling vacant positions (Statistics Canada, 2022). The labor force shortage is a likely obstacle for 35% of businesses, whereas retaining skilled employees is an obstacle for 27.6% of businesses (Statistics Canada, 2022). Labor shortages are costing U.S. businesses approximately $61 billion a month in lost sales (Federal Reserve Bank of Atlanta, 2021). Recruitment and retention issues have never been so acute, and this factor has jeopardized their growth, as well as their obligation to delay or refuse orders, according to the Business Development Bank of Canada (BDC, 2021). Consequently, human resource leaders are tasked with recruiting and retaining employees to deal with this growing challenge by getting on “team employee” and by supporting their wellbeing (Di Meglio, 2021). A way to achieve this task is by relying on HRM practices (Ashton, 2018; Pandita and Ray, 2018; Papa et al., 2018). This is important considering that perceptions of each of the HRM practices by employees were found positively associated with the global perception of HR activities (Cesário, 2015). This highlights the importance of building on a broad set of HRM practices. Faced with international competition and limited financial resources, organizations have every interest in implementing HRM practices that convey a positive image of their organizations (“employer brand”) and ensure that employees are in a state of wellbeing. This strategy not only promotes attraction and retention but also wellbeing, which is a driver of employees’ job performance (Parent-Lamarche et al., 2021; Simard and Parent-Lamarche, 2022). For example, organizational practices of skills development were negatively associated with turnover intentions via their effects on internal employability (i.e., employees’ feeling of having career opportunities in their current job, as well as having value) (Moreira et al., 2022). As mentioned above, from an ethical perspective, performance should not be achieved at the expense of individual wellbeing but should be based on it (Guest, 2017).

However, the dominant theoretical models and empirical research in HRM repeatedly emphasize ways to improve performance through HRM practices from an organizational perspective (Guest, 2017). In this regard, the combination (“bundles”) of HRM practices designed to enhance employees’ performance (e.g., skills and efforts) is called an HPWS (Takeuchi et al., 2007). These HRM practices include, but are not limited to, result-oriented appraisal, selective staffing, extensive skills training, broadened career paths, extensive performance incentives, and internal promotion (Sun et al., 2007). In the field of strategic HRM, HPWS is associated with enhanced levels of performance, both at the organizational and employees’ levels (Den Hartog and Verburg, 2004; Messersmith et al., 2011; Karatepe, 2013; Jackson et al., 2014; Karatepe and Olugbade, 2016; Obeidat et al., 2016; Karadas and Karatepe, 2019). Nonetheless, one of the main criticisms of such an HRM strategic view postulates that employee–employer relationships are conflicting by nature and not congruent, indicating that in such an approach, employee benefices are uncertain (Godard, 2004). In fact, HPWS is likely to result in work intensification, which is a source of stress for employees (Jensen et al., 2013). Furthermore, Han et al. (2020) claimed that despite abundant evidence regarding HPWS’s positive outcomes, its possible negative effects on employees must be examined. In fact, employees’ perspectives and health-related forms of wellbeing (e.g., job stress and burnout) are recurrently considered secondary concerns (Boxall et al., 2016; Peccei and Van De Voorde, 2019). Following these criticisms and concerns for employees, Guest (2017) proposed the integrated mutual gains model as a new analytic framework that suggests that HRM should benefit both employees and organizations. According to the latter statement, HRM practices should be explicitly designed to have a positive impact on wellbeing, which, in turn, can affect performance. Therefore, an “alternative route” to high performance is suggested. This approach differs from standard models in which HRM practices are specifically oriented toward achieving increased performance, considering the issue of wellbeing as a simple collateral effect or a by-product (Guest, 2017). Regardless, several antecedents of wellbeing are actually linked to HRM practices, emphasizing organizations’ responsibility to ensure that their employees’ wellbeing will not erode. This aspect should not be solely based on an ethical argument but also on an economical one, as, for example, absenteeism may be a consequence of low wellbeing (Parent-Lamarche and Laforce, 2022). This concept highlights the “mutual gains” idea since organizations are unlikely to promote wellbeing on ethical grounds alone (Guest, 2017). Based on the previous observations and relying on the analytical framework suggested by Guest (2017), this study aims to build and validate a scale for measuring HRM practices that promote employees’ wellbeing, which further enhances their performance. To our knowledge, at present, no study has attempted to validate this new theoretically proposed analytical approach (i.e., the integrated mutual gains model; Guest, 2017).

### Theoretical background

The integrated mutual gains model builds on the assumptions of social exchange theory (Blau, 1968; Cropanzano and Mitchell, 2005), which states that high employee wellbeing exhibits direct and indirect effects on performance. Moreover, the “happy worker–productive worker” thesis suggests that workers who experience a high level of wellbeing perform well, and vice versa (Warr and Nielsen, 2018). Furthermore, as stated in the theory of resource conservation (Hobfoll, 1989), having an abundance of resources offers a great margin of action to confront future stressful situations while being a predictor of employee wellbeing. This feature enhances willingness to collaborate and achieve (Ryan and Deci, 2000). However, these existing frameworks do not specify a definitive list of HRM practices to guide organizations to ensure that employees will experience high levels of wellbeing and perform well. For example, one of the limitations of the happy worker-productive worker thesis (Wright and Cropanzano, 2000) is that it does not establish the antecedents of such states and thus offers organizations little guidance as to what they can do to promote employee’s wellbeing as well as employee’s performance (Nielsen et al., 2017). To drive employee wellbeing and performance, Guest (2017) suggested emphasizing the five major upstream elements of wellbeing: (1) investing in employees, (2) engaging work (i.e., providing stimulating work), (3) a positive social and physical work environment, (4) voice (i.e., encouraging employee participation), and (5) organizational support. First, it is necessary to assess what is being done in terms of recruitment and selection, as well as training and career development, to invest in employees (Guthrie, 2001; Takeuchi et al., 2007; Ang et al., 2013; Wang et al., 2019). The creation of a stimulating work environment then involves elements of professional challenges in the job, as well as sound information-sharing practices within the organization (Guthrie, 2001; Wang et al., 2019). The social and physical environments are about prioritizing health and safety, providing equal opportunities for everyone in the organization, managing diversity, and implementing place policies against harassment and bullying, as well as ensuring a system of fair collective rewards (Edgar and Geare, 2005; Ang et al., 2013; Wang et al., 2019). Consequently, in terms of encouraging employee participation, this facet is measured by the presence of exhaustive two-way communications (Guthrie, 2001; Jensen et al., 2013; Fu et al., 2015, 2017; Heffernan and Dundon, 2016; Wang et al., 2019). Finally, organizational support includes everything related to flexible and family or personal life-friendly work arrangements (Arthur et al., 2016; Heffernan and Dundon, 2016; Gkorezis et al., 2018; Stirpe et al., 2018). In total, five sets of HRM practices were provisionally outlined and offered as a basis for research to be confirmed, extended, or amended (Guest, 2017).

To answer this call for refocusing HRM research and based on an inventory of existing measurement instruments of HPWS, we proposed a measurement scale integrating the main HRM practices promoting wellbeing, implying the selection and prioritization of existing practices. At present, the nature of HRM practices remains unclear, making it difficult to draw conclusions regarding the kinds of HRM practices that affect employees’ wellbeing (Guest, 2017). In a theoretical manner, Guest (2017) exposed five sets of provisional HRM practices to promote employees’ wellbeing. Therefore, in this study, the appropriate HRM practices considered to provide the necessary resources to promote wellbeing were targeted in accordance with Guest (2017)recommendations. This advancement is important since it is a measurement instrument suited well to the organizational context marked by demands and/or free of resources likely to harm wellbeing, as well as a sense of ethics, which should now be an integral part of the “employer brand,” especially in the context of labor shortages. Our hope is that this scale can respond to the criticism made by Guest (2017), which is aligned with the one previously made by Godard (2004), to the effect that HRM (HPWS) has focused too long on performance and not enough on employee’s wellbeing. It is intended to be more comprehensive than the previous ones by extending practices beyond traditional HRM activities and including practices related, for example, to diversity management.

## Materials and methods

### Development of the scale

An extensive review of the existing literature regarding various HPWS scales was performed. Only scales in English or French were included for item extraction (note that the existing scales listed were all in English). Accordingly, we proceeded to translate these items in accordance with the method proposed by Vallerand (1989). All items from the eligible scales were extracted to create an inventory of items and were then analyzed to verify whether they were adequately formulated to assess the constructs of the integrated model. The scales were further reclassified independently by research team members in each of the five dimensions (provisional sets of HRM practices), as suggested by Guest (2017). All the characteristics of the dimensions studied were considered. More precisely, the provisional HRM practices designed to promote employee wellbeing were consulted to classify each adequate item into the corresponding dimension (i.e., investment in employees, provision of engaging work, positive social and physical environment, voice, and organizational support). Discrepancies in reclassifications were resolved through discussions among team members. To reduce the total number of items to be analyzed for each dimension, we removed duplicates by searching for common phrase structures. Based on existing theoretical and empirical works, a preliminary scale with 66 items was developed from a series of scales validated in the literature (see Supplementary material for the complete list of items in French and in English). The 66 items referred to employees’ perceptions of HRM practices. These perceptions represent both descriptive and evaluative perceptions of HRM practices. Employee reports of HRM practices that are operationalized in the work unit represent descriptive perceptions and are considered implemented HRM practices, as defined by Wright and Boswell (2002). Evaluative perceptions of HRM practices represent interpretations and assessments of the quality of HRM practices, such as their effectiveness and usefulness. For Beijer et al. (2021), descriptive and evaluative items of perceived HRM practices represent related but distinct constructs. As such, both types of items are necessary since they can be expected to be related to different antecedents and have different outcomes.

## Statistical analysis and results

### Validation analysis of the High Wellbeing and Performance Work System Scale

SAS 9.4 (SAS Institute Inc., Cary, NC) was used to analyze the data. Scale (HWBPWS) validation was conducted using exploratory and confirmatory factor analyses (CFA) (Carmines and Zeller, 1979; Churchill, 1979; Fabrigar et al., 1999; O’Rourke and Hatcher, 2013). Exploratory analyses include Cronbach’s alpha for internal consistency and weighted kappa for reliability and principal component analysis (PCA) for factorial structure. In the PCA, varimax rotation was performed to examine the quality of the structure. The number of components was based on theoretical dimensions. Items were considered as loading on a component when their loadings were higher than 0.35. In the CFA of a different sample, maximum likelihood estimation was used. Fit was assessed using three types of indices: an absolute index [standardized root mean square residual (SRMR)], a parsimony index [root mean square error of approximation (RMSEA)], and an incremental index [comparative fit index (CFI)]. The CFA model was further improved according to the modification indices of the first half of the final sample and validated on the remaining half to avoid overfitting. The institutional ethics committee of Université du Québec à Trois-Rivières gave permission to conduct this study.

### Preliminary analyses

First, Cronbach’s alpha and PCA were performed on a first sample of 305 respondents in the initial version of the instrument, including five theoretical dimensions and 66 items. When considering the five theoretical dimensions, Cronbach’s alphas were between 0.41 and 0.83. In the PCA, the five dimensions explained only 37% of the variance, which was deemed insufficient. Instead, by considering the specific HRM-related practices (i.e., dotation, formation, career management, organizational support, autonomy, organizational communication, job security, occupational health and safety, diversity management, compensation management, participation, flexibility, and performance management), assuming 13 dimensions, the Cronbach’s alphas were between 0.23 and 0.88, and 56% of the variance was explained in the PCA. Some items had high loadings in more than one component, whereas other items had not loaded on any component. Furthermore, some components included items that had no sense from a theoretical point of view. Given these results, the organizational support component, as well as other items, was removed, some items were reformulated, and a few more items were added to the second version of the questionnaire. Table 1 shows the respondents’ characteristics for the preliminary analyses (*N* = 305).

**Table 1:** Respondents’ characteristics for preliminary analyses.

Characteristics*	Mean ± Standard deviation or frequency (percentage)
Age (*n* = 298)	42 ± 13
**Sex (*n* = 302)**	
Male	149 (49.34)
Female	153 (50.66)
**Educational level (*n* = 303)**	
None	1 (0.33)
High school	7 (2.31)
Professional school	85 (28.05)
College (general)	37 (12.21)
College (technical)	49 (16.17)
University (undergraduate certificate)	24 (7.92)
University (bachelor’s degree)	74 (24.42)
University (graduate diploma)	7 (2.31)
University (master’s degree)	17 (5.61)
University (doctorate)	2 (0.66)
**Family income (*n* = 298)**	
Less than $20,000	7 (2.35)
$20,000 to $39,999	54 (18.12)
$40,000 to $59,999	80 (26.85)
$60,000 to $79,999	40 (13.42)
$80,000 to $99,999	48 (16.11)
$100,000 to $119,999	27 (9.06)
$120,000 to $139,999	21 (7.05)
$140,000 or more	21 (7.05)
**Employment status (*n* = 303)**	
Non-permanent	25 (8.25)
Permanent	278 (91.75)
**Marital status**	
Single	79 (25.90)
Being part of a couple/married	226 (74.10)
**Parental status (*n* = 300)**	
Without children	167 (55.67)
With dependent child(ren) living with you	133 (44.33)

*N = 305, except when specified in parentheses.

### Test–retest and exploratory analyses

Second, we performed a test–retest; a series of PCAs was then performed, and some items were deleted sequentially. The test–retest enabled us to assess scale reliability to ensure that the respondents understood each item correctly and had consistent answers (Carmines and Zeller, 1979). The second version of the instrument included 60 items and 12 practices (after the preliminary analyses). It was completed by 199 new respondents. Among them, 118 completed the survey twice, with an interval of 14 days. Their demographics are presented in Table 2.

**Table 2:** Demographics of 118 respondents for test–retest.

Characteristics	Mean ± Standard deviation or frequency (percentage)
Age	41 ± 12
**Sex**	
Male	71 (60.17)
Female	47 (39.83)
**Educational level**	
High school	8 (6.78)
Professional school	21 (17.80)
College (general)	7 (5.93)
College (technical)	28 (23.73)
University (undergraduate certificate)	8 (6.78)
University (bachelor’s degree)	27 (22.88)
University (graduate diploma)	5 (4.24)
University (master’s degree)	13 (11.02)
University (doctorate)	1 (0.85)
**Family income**	
Less than $20,000	1 (0.85)
$20,000 to $39,999	5 (4.24)
$40,000 to $59,999	18 (15.25)
$60,000 to $79,999	10 (8.47)
$80,000 to $99,999	33 (27.97)
$100,000 to $119,999	16 (13.56)
$120,000 to $139,999	8 (6.78)
$140,000 or more	27 (2.88)
**Employment status**	
Full time	118 (100.00)
**Marital status**	
Single	44 (37.29)
Being part of a couple/married	74 (62.71)
**Parental status**	
Without children	70 (59.32)
With dependent child(ren) living with you	48 (40.68)

By utilizing only the data of 118 workers who completed the survey twice in the test–retest, the weighted kappa measures were calculated. Items with acceptable kappa values (0.40–0.78) were kept for the next step, whereas two items (formation2 and formation3) were excluded because they had poor kappa values (below 0.40).

Internal consistency and factorial structure were examined on the whole sample of *N* = 199. Table 3 shows the demographics of 199 respondents for the test, including those from the test–retest.

**Table 3:** Demographics of 199 respondents for test (including those from test–retest).

Characteristics	Mean ± Standard deviation or frequency (percentage)
Age	41 ± 12
**Sex**	
Female	88 (44.22)
Male	111 (55.78)
**Educational level**	
High school	20 (10.05)
Professional school	25 (12.56)
College (general)	11 (5.53)
College (technical)	53 (26.63)
University (undergraduate certificate)	12 (6.03)
University (bachelor’s degree)	49 (24.62)
University (graduate diploma)	9 (4.52)
University (master’s degree)	18 (9.05)
University (doctorate)	2 (1.01)
**Family income**	
Less than $20,000	3 (1.51)
$20,000 to $39,999	10 (5.03)
$40,000 to $59,999	31 (15.58)
$60,000 to $79,999	23 (11.56)
$80,000 to $99,999	51 (25.63)
$100,000 to $119,999	27 (13.57)
$120,000 to $139,999	15 (7.54)
$140,000 or more	39 (19.60)
**Employment status**	
Full time	199 (100.00)
**Marital status**	
Single	70 (35.18)
Being part of a couple/married	129 (64.82)
**Parental status**	
Without children	106 (53.27)
With dependent child(ren) living with you	93 (46.73)

During the four iteration rounds, items were sequentially removed when they lowered the internal consistency, loaded on more than one component or not loaded on any component, or when an item was not in a component that made sense from a theoretical perspective. The items removed in each of the three first steps were as follows: (1) formation1, career2, communication1, security1, security2, security3, and compensation3; (2) communication2, participation1–4, and flexibility1; and (3) compensation1, flexibility2, and flexibility8. In the first step, the entire job security dimension was removed, whereas the compensation management dimension was split into three dimensions: indirect compensation (also termed employee benefits), performance compensation, and equity compensation. In the second step, the entire participation dimension was removed. In the third step, both autonomy and organizational communication dimensions were combined into a single dimension. The final version obtained in the fourth round included 42 items and 11 HRM practices (dimensions), which explained 79% of the variance in the PCA. The final solution seemed acceptable for the test sample. Table 4 shows the Cronbach’s alphas of HRM practices (dimensions) in the last step of the test data (*N* = 199).

**Table 4:** Cronbach’s alphas of human resource management practices/dimensions at the last step on test data (*N* = 199).

Component/Dimension	# items	Cronbach’s alphas
Dotation	3	0.87
Formation	4	0.89
Career management	3	0.77
Autonomy and organizational communication	5	0.91
Occupational health and safety	4	0.92
Diversity management	4	0.91
Indirect compensation	3	0.86
Performance compensation	3	0.81
Equity compensation	2	0.52
Flexibility	5	0.85
Performance management	6	0.95

The correlation coefficients of HRM practices/dimensions at the last step on test data (*N* = 199) are presented in Table 5.

**Table 5:** Correlation matrix of human resource management practices/dimensions at the last step on test data (*N* = 199).

	1	2	3	4	5	6	7	8	9	10	11
1. Dotation	1.00	0.42***	0.48***	0.57***	0.52***	0.63***	0.28***	0.19**	0.38***	0.27***	0.31***
2. Formation		1.00	0.61***	0.48***	0.35***	0.43***	0.42***	0.31***	0.29***	0.31***	0.59***
3. Career management			1.00	0.58***	0.44***	0.46***	0.31***	0.31***	0.35***	0.33***	0.51***
4. Autonomy and organizational communication				1.00***	0.65***	0.62***	0.38***	0.23**	0.37***	0.25**	0.35***
5. Occupational health and safety					1.00	0.66***	0.44***	0.17*	0.32***	0.23**	0.35***
6. Diversity management						1.00	0.44***	0.12	0.38***	0.29***	0.37***
7. Indirect compensation							1.00	0.02	0.32***	0.15*	0.43***
8. Performance compensation								1.00	−0.09	0.38***	0.48***
9. Equity compensation									1.00	0.10	0.19**
10. Flexibility										1.00	0.47***
11. Performance management											1.00

***p < 0.0001; **p < 0.01; *p < 0.05.

Consequently, CFA aimed to confirm the quality of the factor structure of the scale generated in the previous step. CFA was performed on a new sample to test the structure retained in the PCA. The sample was divided randomly into two parts: two-thirds (*N* = 598) were used as a training set, and the last third (*N* = 398) was used to validate the final model. Table 6 shows the demographics of respondents for the CFA (training set), and Table 7 presents the demographics of respondents for the final CFA (validation set).

**Table 6:** Demographics of respondents for the confirmatory factor analyses (training set).

Characteristics*	Mean ± Standard deviation or frequency (percentage)
Age	40 ± 12
**Sex**	
Male	260 (43.48)
Female	338 (56.52)
**Educational level**	
None	1 (0.17)
High school	47 (7.86)
Professional school	56 (9.36)
College (general)	51 (8.53)
College (technical)	143 (23.91)
University (undergraduate certificate)	43 (7.19)
University (bachelor’s degree)	167 (27.93)
University (graduate diploma)	21 (3.51)
University (master’s degree)	60 (10.03)
University (doctorate)	9 (1.51)
**Family income**	
Less than $20,000	5 (0.84)
$20,000 to $39,999	38 (6.35)
$40,000 to $59,999	102 (17.06)
$60,000 to $79,999	94 (15.72)
$80,000 to $99,999	89 (14.88)
$100,000 to $119,999	99 (16.56)
$120,000 to $139,999	58 (9.70)
$140,000 or more	113 (18.90)
**Employment status**	
Full time	598 (100.00)
**Marital status**	
Single	189 (31.61)
Being part of a couple/married	409 (68.39)
**Parental status**	
Without children	353 (59.03)
With dependent child(ren) living with you	245 (40.97)

*n = 598.

**Table 7:** Demographics of respondents for final confirmatory factor analyses (validation set).

Characteristics*	Mean ± Standard deviation or frequency (percentage)
Age	40 ± 12
**Sex**	
Male	177 (44.47)
Female	221 (55.53)
**Educational level**	
None	2 (0.50)
High school	36 (9.05)
Professional school	45 (11.31)
College (general)	32 (8.04)
College (technical)	95 (23.87)
University (undergraduate certificate)	25 (6.28)
University (bachelor’s degree)	114 (28.64)
University (graduate diploma)	17 (4.27)
University (master’s degree)	29 (7.29)
University (doctorate)	3 (0.75)
**Family income**	
Less than $20,000	2 (0.50)
$20,000 to $39,999	28 (7.04)
$40,000 to $59,999	42 (10.55)
$60,000 to $79,999	56 (14.07)
$80,000 to $99,999	64 (16.08)
$100,000 to $119,999	74 (18.59)
$120,000 to $139,999	54 (13.57)
$140,000 or more	78 (19.60)
**Employment status**	
Full time	398 (100.00)
**Marital status**	
Single	114 (28.64)
Being part of a couple/married	284 (71.36)
**Parental status**	
Without children	231 (58.04)
With dependent child(ren) living with you	167 (41.96)

*n = 398.

The adjustment of the model obtained in the PCA had an inadequate fit on the training sample of *N* = 598, as shown in Table 8. Even if CFI and TLI showed an adequate fit, SRMR was not ideal, and RMSEA and GFI suggested only a fair fit.

**Table 8:** Goodness-of-fit indices of confirmatory factor analyses.

Sample	Training	Training	Validation
Model	Exploratory	Final	Final
**Statistic**			
X^2^	2252.43	1336.06	1139.75
Degrees of freedom	764	549	549
SRMR	0.0630	0.0508	0.0503
RMSEA (90% CI)	0.0571 (0.0544–0.0599)	0.0490 (0.0457–0.0523)	0.0521 (0.0478–0.0563)
CFI	0.9078	0.9443	0.9403
TLI	0.8961	0.9360	0.9315
GFI	0.8289	0.8819	0.8640

SRMR, standardized root mean square residual; RMSEA, root mean square error of approximation; CFI, comparative fit index; TLI, Tucker-Lewis Index; GFI, Goodness of Fit Index.

Using the modification indices, which indicated the items that should be excluded to improve overall fit, six items (i.e., communication3, communication4, diversity1, flexibility7, compensation5, and compensation9) were further removed to obtain a final model with 36 items. Ten dimensions were left since the equity compensation dimension was removed, and the organizational communication items were removed from the autonomy dimension. The removal of these items and dimensions based on modification indices improved the overall fit. Table 9 shows the list of final items and dimensions.

**Table 9:** List of final items and dimensions of the “High Wellbeing and Performance Work System Scale” (French version available in Supplementary material).

Items	Dimensions/Practices
Are the recruitment and selection processes in this organization impartial (fair and equitable)? (dotation1)	Dotation
Is favoritism not evident in any of the recruitment decisions made here? (dotation2)	
Does this organization not need to pay more attention on the way it recruits people? (dotation3)	
Are extensive training programs provided for me? (formation4)	Formation
Will I normally undergo training programs every year? (formation5)	
Are formal training programs available to teach newly hired personnel the skills they need to perform their jobs? (formation6)	
Are formal training programs offered to me to increase my promotability in this organization? (formation7)	
Do I have a clear career path (planned promotions) within the organization? (career1)	Career management
Are my career aspirations (promotions and/or skills development envisaged within the company) known by my immediate supervisors? (career3)	
Do I have more than one potential position for promotion? (career4)	
Do I have several opportunities to decide how to do my work? (autonomy1)	Autonomy
If a problem emerges with my work, can I take actions to remedy it? (autonomy2)	
Will my organization give me autonomy to make decisions in the context of my work? (autonomy3)	
Is my work environment safe? (ohs1)	Occupational health and safety
Has my health not suffered due to working for this organization? (ohs2)	
Do I always feel safe working in these conditions? (ohs3)	
Does this organization do what it can to ensure the wellbeing of its employees? (e.g., health/wellness committee) (ohs4)	
Do I feel that management is supportive of cultural differences in this organization? (diversity2)	Diversity management
Do I feel that men and women have the same employment opportunities in this organization? (diversity3)	
Do I feel that equal employment opportunity is promoted in this organization? (diversity4)	
Is a part of my compensation/salary based on how well the organization is doing financially? (compensation2)	Performance compensation
Is a part of my compensation/salary based on how well my workgroup or department performs? (compensation4)	
Does a part of my compensation/salary depend on my individual work performance? (compensation10)	
Does my organization offer me a retirement plan (pension fund, etc.)? (compensation6)	Indirect compensation
Does my organization offer me benefits that meet my expectations and needs? (compensation7)	
Does my organization provide me with insurance coverage (e.g., drug, dental, life insurance, etc.)? (compensation8)	
Does this organization allow job-sharing schemes (sharing a full-time job with another employee)? (flexibility3)	Flexibility
Do I have the ability to reduce working hours (e.g., switching from full-time to part-time employment)? (flexibility4)	
Does this organization allow compressed hours (i.e., working standard hours across fewer days)? (flexibility5)	
Do I have the ability to change set working hours (including shift pattern)? (flexibility6)	
Do I receive formal performance appraisals or evaluation on a routine basis? (perfomance1)	Performance management
Do I receive formal performance feedback from more than one sources (i.e., feedback from several individuals such as supervisors, peers, etc.)? (performance2)	
Are my performance appraisals based on objective, quantifiable results? (performance3)	
Do my performance appraisals include management by objective with mutual goal setting? (performance4)	
Do my performance appraisals include developmental feedback? (performance5)	
Are performance appraisals used to plan skill development and training for future advancements? (performance6)	

^a^The English version was not validated. Only the French version was validated in this study.

^b^A seven-point scale with responses to each item ranging from 1 to 7 was used: 1 (strongly disagree); 2 (disagree); 3 (somewhat disagree); 4 (neither agree nor disagree); 5 (somewhat agree); 6 (agree); and 7 (strongly agree).

This model was tested in the validation set and maintained a proper fit (see Table 7). Cronbach’s alphas for the CFA samples are shown in Table 10.

**Table 10:** Cronbach’s alphas for confirmatory factor analysis samples.

Sample	Training		Training		Validation	
Model	Exploratory		Final		Final	
Component	# items	Alphas	# items	alphas	# items	Alphas
Dotation	3	0.83	3	0.83	3	0.84
Formation	4	0.89	4	0.89	4	0.88
Career management	3	0.75	3	0.75	3	0.78
Autonomy/Organizational communication	5	0.87	3	0.88	3	0.89
Occupational health and safety	4	0.89	4	0.89	4	0.90
Diversity management	4	0.87	3	0.86	3	0.90
Indirect compensation	3	0.84	3	0.84	3	0.84
Performance compensation	3	0.83	3	0.83	3	0.85
Equity compensation	2	0.45	0	NA	0	NA
Flexibility	5	0.78	4	0.79	4	0.83
Performance management	6	0.94	6	0.94	6	0.94

*NA, not available.

The properties of the revised CFA model on the validation sample are presented in Table 11. In addition, Cronbach’s coefficients for the final dimensions varied from 0.79 (career management) to 0.94 (performance management). All items were statistically significant.

**Table 11:** Properties of the final confirmatory factor analysis model on the validation sample.

Construct and indicators	Standardized loading	Indicator reliability	Error variance	*t*	Construct reliability	Variance extracted estimate
Dotation					0.84	0.64
dotation1	0.83	0.69	0.31	36.60		
dotation2	0.82	0.67	0.33	35.21		
dotation3	0.74	0.55	0.45	26.56		
Formation					0.88	0.65
formation4	0.82	0.68	0.32	41.86		
formation5	0.74	0.55	0.45	28.91		
formation6	0.77	0.60	0.40	33.24		
formation7	0.89	0.78	0.22	56.79		
Career management					0.79	0.56
career1	0.83	0.69	0.31	37.43		
career3	0.59	0.34	0.66	15.81		
career4	0.80	0.65	0.35	34.29		
Autonomy					0.89	0.73
autonomy1	0.84	0.71	0.29	43.73		
autonomy2	0.85	0.73	0.27	46.39		
autonomy3	0.87	0.76	0.24	50.02		
Occupational health and safety					0.91	0.71
ohs1	0.85	0.72	0.28	50.85		
ohs2	0.80	0.63	0.37	38.50		
ohs3	0.94	0.88	0.12	85.68		
ohs4	0.78	0.61	0.39	35.78		
Diversity management					0.90	0.75
diversity2	0.82	0.67	0.33	41.58		
diversity3	0.86	0.73	0.27	50.43		
diversity4	0.92	0.84	0.16	69.91		
Performance compensation					0.85	0.66
compensation2	0.72	0.51	0.49	24.92		
compensation4	0.92	0.85	0.15	47.73		
compensation10	0.80	0.63	0.37	32.87		
Indirect compensation					0.84	0.64
compensation6	0.76	0.57	0.43	27.27		
compensation7	0.83	0.68	0.32	33.60		
compensation8	0.81	0.65	0.35	31.90		
Flexibility					0.83	0.55
flexibility3	0.59	0.35	0.65	16.00		
flexibility4	0.72	0.51	0.49	24.48		
flexibility5	0.79	0.63	0.37	32.47		
flexibility6	0.84	0.70	0.30	37.71		
Performance management					0.94	0.74
performance1	0.79	0.63	0.37	39.90		
performance2	0.78	0.61	0.39	36.83		
performance3	0.87	0.76	0.24	63.77		
performance4	0.89	0.80	0.20	75.34		
performance5	0.92	0.85	0.15	96.99		
performance6	0.89	0.80	0.20	75.88		

The mean scores and standard deviation (SD) for each dimension of the validation set (*N* = 398) are presented in Table 12. The diversity management dimension showed a high mean score (5.21, *SD* = 1.41), whereas the performance compensation dimension showed a low mean score (3.02, *SD* = 1.75).

**Table 12:** Mean scores for each dimension on the validation set (*N* = 398).

Dimension	Mean	Standard deviation
Dotation	4.26	1.45
Formation	3.80	1.60
Career management	3.74	1.52
Autonomy	4.86	1.42
Occupational health and safety	5.16	1.42
Diversity management	5.21	1.41
Performance compensation	3.02	1.75
Indirect compensation	5.01	1.71
Flexibility	3.11	1.63
Performance management	3.73	1.69

The correlation coefficients for each dimension on the validation set (*N* = 398) are presented in Table 13.

**Table 13:** Correlation matrix for each dimension on the validation set (*N* = 398).

	1	2	3	4	5	6	7	8	9	10
1. Dotation	1	0.36***	0.43***	0.39***	0.52***	0.52***	0.25***	0.21***	0.27***	0.37***
2. Formation		1	0.60***	0.27***	0.29***	0.40***	0.34***	0.23***	0.36***	0.53***
3. Career management			1	0.45***	0.42***	0.50***	0.28***	0.34***	0.41***	0.57***
4. Autonomy				1	0.50***	0.52***	0.20***	0.19**	0.30***	0.35***
5. Occupational health and safety					1	0.58***	0.27***	0.21***	0.33***	0.43***
6. Diversity management						1	0.34***	0.09	0.29***	0.46***
7. Indirect compensation							1	0.04	0.16**	0.40***
8. Performance compensation								1	0.39***	0.42***
9. Flexibility									1	0.58***
10. Performance management										1

***p < 0.0001; **p < 0.01; *p < 0.05.

The convergent and divergent validity scores for each dimension on the validation set (*N* = 398) are presented in Table 14.

**Table 14:** Convergent and divergent validity scores for each dimension on the validation set (*N* = 398).

Dimension	# items	Convergent validity*	Divergent validity*	Success	Success rate
Dotation	3	0.67;0.73	0.13;0.52	27/27	100%
Formation	4	0.71;0.79	0.13;0.62	36/36	100%
Career management	3	0.54;0.68	0.21;0.60	26/27	96%
Autonomy	3	0.77;0.80	0.15;0.56	27/27	100%
Occupational health and safety	4	0.74;0.87	0.13;0.61	36/36	100%
Diversity management	3	0.76;0.83	0.06;0.56	27/27	100%
Indirect compensation	3	0.67;0.72	−0.12;0.41	27/27	100%
Performance compensation	3	0.66;0.79	−0.02;0.42	27/27	100%
Flexibility	4	0.54;0.70	0.05;0.54	36/36	100%
Performance management	6	0.76;0.88	0.25;0.55	54/54	100%

*Range of Pearson’s correlations.

## Discussion

This study established the reliability and validity of a new scale (HWBPWS) for evaluating HRM practices conducive to both employees’ wellbeing and performance. The development and validation of such an instrument is important at the theoretical, empirical, and practical levels, as argued by many authors for additional research on this topic (e.g., Peccei and Van De Voorde, 2019; Beijer et al., 2021). Indeed, there is a lack of consensus and clarity in the literature surrounding both the conceptualization and measurement of HRM practices and systems (Boon et al., 2019; Peccei and Van De Voorde, 2019; Beijer et al., 2021), making new research on this topic central to better understand the link between HRM-wellbeing and performance. To date, the dominant theoretical models and empirical research in HRM have repeatedly emphasized ways to improve performance through HRM practices from an organizational perspective (Guest, 2017). Therefore, Guest (2017) developed a new analytic framework that suggests that HRM should benefit both employees and organizations. The main underlying assumption is that HRM practices should be explicitly designed to have a positive impact on employees’ wellbeing, which, in turn, can affect their performance. To our knowledge, at present, no study has attempted to validate this new theoretically proposed analytical approach (i.e., the integrated mutual gains model). However, the validation of this approach can ensure the new orientation of empirical research and ultimately the renewal of HRM practices within organizations. Such a renewal that implies placing employees at the heart of organizations (employee perspective instead of organizational perspective) appears to be necessary not only from an ethical point of view but also in the context of major labor shortages.

First, an extensive review of the literature on scales that used HPWS to assess HRM practices, as well as an extraction of items related to the theoretical dimensions of the integrated mutual gains model, were performed. These theoretical dimensions are as follows: (1) investing in employees, (2) engaging work (i.e., providing stimulating work), (3) a positive social and physical work environment, (4) voice (i.e., encouraging employee participation), and (5) organizational support. Based on these preliminary steps, an initial scale with the most relevant items was developed. In total, 66 items were initially extracted from the literature. The results regarding the five theoretical dimensions were inadequate, which led to validation based on the specific HRM practices (i.e., dotation, formation, career management, organizational support, autonomy, organizational communication, job security, occupational health and safety, diversity management, compensation management, participation, flexibility, and performance management) included in these five initial theoretical dimensions, assuming 13 dimensions. At this stage, the analyses revealed some abnormalities; therefore, the organizational support dimension and other items were removed. Some items were reformulated, and a few more were added to the second version of the questionnaire. Notably, the organizational support dimension comprised only two items, whereas the suggested minimum requirement was three items (O’Rourke and Hatcher, 2013).

Second, a test–retest, with an interval of 14 days (Carmines and Zeller, 1979), followed by a series of PCAs, was performed, and some items were then deleted sequentially. Two items (formation2 and formation3) were excluded before the four iteration rounds because they were not reliable and had poor kappa values (below 0.40). These items were not well understood by the respondents. This factor might be because they approached the notions of generic and specific skills (i.e., “I have received intensive/extensive training in company-specific skills” and “I have received intensive/extensive training in generic skills”). These specific formation practices were probably too specific and were not necessarily generalized among organizations. Also, it should be noted that it is important to create a stimulating learning environment in which employees’ actual participation in skills development is supported by organizations (De Vos et al., 2011). In contrast, the remaining items related to formation appeared highly generalizable. The second version of the instrument included 60 items and 12 practices (dimensions). During the four iteration rounds, the items were sequentially removed (i.e., formation1, career2, communication1, security1, security2, security3, compensation3, communication2, participation1 to 4, flexibility1, compensation1, flexibility2, and flexibility8). The entire job security dimension and participation dimension were removed, whereas the compensation management dimension was split into three dimensions: indirect compensation (also termed employee benefits), performance compensation, and equity compensation. In addition, both the autonomy and organizational communication dimensions were combined into a single dimension. Although combining these two practices (dimensions) seemed counterintuitive, this dimension remains in line with the provisional five dimensions suggested by Guest (2017). Indeed, these two dimensions consisted of engaging work by providing a stimulating initial work dimension. As mentioned above, the creation of a stimulating work environment involves elements of professional challenges in the job, as well as sound information-sharing practices within the organization (Guthrie, 2001; Wang et al., 2019). Moreover, the management of total compensation is broad and may include distinct elements, such as direct and indirect compensations (Perkins and Jones, 2020). In addition, various forms of equity, such as internal (i.e., in comparison with colleagues inside the organization), external (i.e., in comparison with compensation offered in other organizations), and individual equities (i.e., in coherence with employees’ efforts and/or performance), are essential to compensation management (Hallée et al., 2021). Therefore, the analyses unsurprisingly led to three distinct dimensions reflecting these elements. Furthermore, the participation dimension appeared to be eclectic in the sense that it comprised items related to quality circles or problem-solving groups and access to a formal grievance procedure, as well as routinely administered attitude surveys. These items initially comprised the voice provisional dimension. Therefore, the final version obtained at this stage included 42 items and 11 HRM practices (dimensions), which seemed acceptable.

Finally, CFAs were performed on a new sample divided randomly into two parts (i.e., training set and validation set) to confirm the quality of the factor structure of the scale generated by the PCA. Using the modification indices, six items (i.e., communication3, communication4, diversity1, flexibility7, compensation5, and compensation9) were removed, resulting in a 36-item instrument for measuring 10 HRM practices. The equity compensation dimension was removed, and organizational communication items were excluded from the autonomy dimension. Although equity is an essential element of compensation management (Hallée et al., 2021), the three distinct forms of equity may not be coherent. However, each form of equity should be measured separately. This approach seems consistent with the fact that all forms of equity are difficult to optimize simultaneously, as they are often in contradiction. On the one hand, if an organization favors external equity, negative repercussions on internal equity perceptions may arise (St-Onge, 2020). On the other hand, if an organization favors internal equity, more competent and performant employees may perceive inequity. Regarding communication items, even though they were related to autonomy, as shown in the PCA analyses, the CFA analyses confirmed that this structure could not be maintained.

### Implications, future research, and limitations

One novelty of the scale developed in this study is the inclusion of and focus on HRM practices that may improve employees’ wellbeing and, consequently, their job performance. These HRM practices are considered antecedents of employees’ wellbeing, according to the integrated mutual gains model developed by Guest (2017) as well as the mutual gains’ perspective in general. Although the five provisional sets of practices were not validated as such, the practices that emerge from them are assembled into alternative sets of practices. These sets of practices reflect HRM activities that are considered conducive to employees’ wellbeing and, consequently, their job performance. That said, the fact that the five theoretical dimensions proposed by Guest (2017) have not been validated as such opens the way to the study of alternative possibilities, such as conflicting outcomes and mutual losses’ perspectives. For many years now, skeptics of mutual gain perspectives have raised concerns that the benefits of HRM practices go only in one direction—that is, toward organizational performance at the expense of employee health (e.g., Ogbonnaya et al., 2017). They argue that HRM practices are used primarily to drive organizational performance, creating work overload for employees. In their systematic review and meta-analysis of workplace resources to improve both employee wellbeing and performance, Nielsen et al. (2017) found meta-analytic support for the mutual gain perspective. Although the perspective of mutual gains seems to be the most promising and ideal, reality seems more complex. Therefore, it is possible that HRM practices can hardly be grouped into practices aimed exclusively at the wellbeing of employees. Instead, it is possible that some specific HRM practices may have a negative effect on wellbeing, and others may have a positive effect. Therefore, future empirical research should consider possible trade-offs as well as potential bundles of HRM practices that could be considered resources (leading to win–win situations) and other bundles that could be considered demands (leading to win–lose or lose–lose situations). Furthermore, future research is necessary to evaluate the predictive capacity of this new scale. Further longitudinal research should verify whether the 10 HRM practices included in the HWBPWS are conducive to employees’ wellbeing, which later translates into job performance. In addition, although employees’ perspectives seem to be the most appropriate for measuring HRM practices (Beijer et al., 2021), future studies could try to adapt the HWBPWS for managers. Indeed, a combination of perspectives could help improve the implementation of HRM practices, the uniformized perception across employees, and their effects on outcomes. Moreover, understanding the conditions under which congruent or noncongruent perceptions of HRM practices between employees and managers take place could provide useful avenues for future research (Wong and Giessner, 2018; Beijer et al., 2021). In addition, the differentiated effects of these 10 HRM practices should be determined according to the sectors and the size of the organizations, as well as according to the age, gender, and personality of the employees. As such, specific sets of practices could be valued and promoted in organizations depending on those variables. This concept is possible because the best HRM practices conducive to wellbeing could be those that are adapted to organizations and employees’ specificities. From a practical point of view, this could allow organizations to orient their HRM practices differently and in a more enlightened way. In practice, this might result in the necessity to implement different practices for different organizations and for the different segments of employees within them. By focusing first on the wellbeing of their employees, organizations should gain attraction, retention, and performance, which is a considerable advantage, if not a necessity, in the context of labor shortages. Therefore, this should consolidate a win–win situation for both employees and organizations and thus support the integrated mutual gains model.

This study has some limitations. First, self-reported data could lead to common method variance problems. Second, although we conducted a review of the instruments in the literature, a systematic review as such, and above all, was not performed. We listed only the instruments that were available to the readers. Consequently, we missed relevant items. Third, to develop and validate the HWBPWS scale, we relied exclusively on a review of the instruments available in the literature (deductive approach) and were unable to complete it with a qualitative analysis (inductive approach). The inductive approach would have allowed us to extract relevant information through focus group discussions and interviews with key respondents, such as HRM managers and employees, to identify appropriate items that could have been added. Fourth, while our scale has a strong internal structure, excluding items that did not contributed to this structure might have led to the neglect of important dimensions. In this regard, an inductive approach combined with a deductive one could have prevented it and ensured a wider and more accurate list of items and dimensions. For instance, organizational communication, job security, and participation had to be removed from the final list of dimensions. This may have been caused by the samples used in our study. Further studies should verify if it is possible to reintegrate them into the new scale “HWBPWS” in a revisited version of it. Please note that all items related to those dimensions are presented in Supplementary material. Fifth, the scale was validated in French (although the English version is presented in this paper), indicating that further work should validate it in other cultures and languages.

## Conclusion

In summary, the objective of this study was to elaborate on and validate a more complete scale for measuring HRM practices based on existing scales (HPWS) by prioritizing elements related to wellbeing; therefore, a new scale, HWBPWS, was created. Exploratory and confirmatory factor analyses were carried out to achieve this objective. The analyses revealed a 36-item instrument for measuring 10 HRM items. To our knowledge, this study was the first to verify the empirical validation of the new theoretically proposed analytical approach suggested by Guest (2017). Although the five provisional sets of practices of the integrated mutual gains model were not validated as such, the practices that emerged from them were assembled into alternative sets of practices considered conducive to employees’ wellbeing and, consequently, their job performance. Therefore, the new orientation of empirical research and, ultimately, the renewal of HRM practices within organizations from the employee’s perspective instead of the organizational perspective was partially supported. This aspect was necessary not only from an ethical point of view but also in the context of major labor shortages. That said, it is important to underline the fact that it might be reasonable to expect that items constructed and selected by other researchers to support organizational performance will not necessarily support employees’ wellbeing. The initial idea of our study was to highlight existing and widely documented practices in the HPWS literature and identified those that aligned with Guest’s proposed dimensions to create a new scale that could both be conducive to employees’ wellbeing and performance. Considering that the five dimensions proposed by Guest (2017) could not be confirmed in this study, it would be preferable for future studies to try to improve our new scale by proposing practices that are not typically included in the traditional HPWS scales and with an alternative methodology. There is room to imagine inventories of HRM practices and to ask employees if they are each part of a strategy of mutual gain or of negative interdependence between wellbeing and performance. Possible paradoxical effects of HRM practices included in the new HWBPWS scale should be highlighted. For example, pay-for-performance may seem fair and conducive to both wellbeing and performance. But it is alternatively possible that this can lead to exhaustion in the long run. Special attention should be paid to this in future studies that will focus on the predictive capacity of the scale. In particular, it will be possible to verify whether the effect of certain HRM practices can be influenced by employees’ individual characteristics (e.g., personality traits, emotional intelligence, humility). Also, diversity may seem fair, but to have the desired effects on wellbeing and performance, it is possible that employees will need to perceive that it is achieve with noble intentions and not just for the sole purpose of filling the workforce or to project a good corporate image. Additionally, and as previously mentioned further research is necessary to validate the predictive capacity of this new scale and to consider alternative perspectives regarding the possible effects of HRM practices. In this regard, its impact in terms of wellbeing, job satisfaction, exhaustion or instrumentalization (dehumanization) could be explored. Also, it would be possible to verify whether HRM practices predict wellbeing and performance via perceived social support or whether social support accentuates the effect of HRM practices on these outcomes. This research was a first step, requiring long-term work with multiple iterative steps, with the main intention of placing employees’ wellbeing at the heart of organizations.

## Data availability statement

The raw data supporting the conclusions of this article will be made available by the authors, without undue reservation.

## Ethics statement

The studies involving human participants were reviewed and approved by the Institutional Review Board of the Université du Québec à Trois-Rivières. The patients/participants provided their written informed consent to participate in this study.

## Author contributions

AP-L and JD-G designed the study. AP-L was responsible for the data collection and conducted the review of the literature and drafted the manuscript. A-SJ carried out the analysis. A-SJ, AP-L, and JD-G interpreted the results. JD-G and A-SJ provided critical feedback for the manuscript. All authors approved the final manuscript and gave their consent.

## Conflict of interest

The authors declare that the research was conducted in the absence of any commercial or financial relationships that could be construed as a potential conflict of interest.

## Publisher’s note

All claims expressed in this article are solely those of the authors and do not necessarily represent those of their affiliated organizations, or those of the publisher, the editors and the reviewers. Any product that may be evaluated in this article, or claim that may be made by its manufacturer, is not guaranteed or endorsed by the publisher.
