# Supplementary material for: Toward a new model of human resource management practices: construction and validation of the High Wellbeing and Performance Work System Scale
Source: Front Psychol. 2023 May 10;14:1151781. doi: 10.3389/fpsyg.2023.1151781 (PMC10205990; doi:10.3389/fpsyg.2023.1151781)
Supplement: Supplementary file 1 [file Data_Sheet_1.docx]

| *Supplementary materials:*  *List of final items and dimensions of the “High Well-Being and Performance Work System Scale, HWBPWS” in French* | |
| --- | --- |
| Items | Dimensions/Practices |
| Les processus de recrutement et de sélection de cette organisation sont impartiaux (justes et équitables)? (dotation1) | Dotation |
| Il ne semble pas y avoir de favoritisme dans aucune des décisions de recrutement prises ici? (dotation2) |  |
| Cette organisation n'a pas besoin de prêter plus d'attention à la façon dont elle recrute des employés? (dotation3) |  |
| Des programmes de formation approfondis me sont offerts? (formation4) | Formation |
| Je vais normalement suivre des programmes de formation tous les ans? (formation5) |  |
| Il existe des programmes de formation officiels pour enseigner aux nouveaux employés les compétences dont ils ont besoin pour effectuer leur travail? (formation6) |  |
| Des formations me sont proposées afin d’accroître mes possibilités d’avancement/promotion dans cette organisation? (formation7) |  |
| J'ai un cheminement de carrière (promotions envisagées) clair au sein de l'organisation? (career1) | Gestion de carrière |
| Mes aspirations professionnelles (promotions et/ou développement des compétences envisagées) au sein de l'organisation sont connues de mes supérieurs immédiats? (career3) |  |
| J’ai plusieurs possibilités de promotion au sein de cette organisation? (career4) |  |
| J'ai beaucoup d'occasions de décider comment faire mon travail? (autonomy1) | Autonomie |
| Si un problème survient dans mon travail, je peux prendre des mesures pour y remédier? (autonomy2) |  |
| Mon organisation me donne de l’autonomie pour prendre des décisions dans le cadre de mon travail? (autonomy3) |  |
| Mon environnement de travail est sécuritaire? (ohs1) | Santé et sécurité au travail |
| Ma santé ne s’est pas détériorée du fait de travailler pour cette organisation? (ohs2) |  |
| Je me sens toujours en sécurité en travaillant ici dans ces conditions? (ohs3) |  |
| Cette organisation fait tout ce qu'elle peut pour assurer la sécurité et le bien-être de ses employés? (ex: comité SST et/ou santé/mieux-être) (ohs4) |  |
| Je pense que la direction soutient la différence culturelle dans cette organisation? (diversity2) | Gestion de la diversité |
| Je pense que les hommes et les femmes ont les mêmes opportunités d’emploi dans cette organisation? (diversity3) |  |
| Je pense que l'égalité des chances dans l’accès à l'emploi est facilitée dans cette organisation? (diversity4) |  |
| Une partie de ma rémunération dépend de la situation financière de l’organisation? (compensation2) | Rémunération selon la performance |
| Une partie de ma rémunération est basée sur les performances de mon équipe de travail ou de mon département? (compensation4) |  |
| Une partie de mon salaire dépend de ma performance individuelle de travail? (compensation10) |  |
| Mon organisation m’offre un régime de retraite (fonds de pension, RVER, REER collectif, etc.)? (compensation6) | Rémunération indirecte |
| Mon organisation m’offre des avantages sociaux qui correspondent à mes attentes et à mes besoins? (compensation7) |  |
| Mon organisation m’offre une couverture d’assurance (ex : médicaments, dentaires, assurance vie, etc.) (compensation8) |  |
| Cette organisation permet le partage d’emploi (partage d’un emploi à temps plein avec un autre employé)? (flexibility3) | Flexibilité |
| J'ai la capacité de réduire mes heures de travail (par exemple, passer d'un emploi à temps plein à un emploi à temps partiel)? (flexibility4) |  |
| Cette organisation permet de comprimer ses heures de travail (c.-à-d. des heures standards travaillées sur moins de jours)? (flexibility5) |  |
| J'ai la possibilité de changer les heures de travail définies (y compris l’horaire de travail)? (flexibility6) |  |
| Je reçois régulièrement des évaluations formelles de performance? (perfomance1) | Gestion de la performance |
| Je reçois de la rétroaction formelle provenant de plusieurs sources (des superviseurs, des collègues, etc.)? (performance2) |  |
| Mon évaluation de performance est basée sur des résultats objectifs et quantifiables? (performance3) |  |
| Mon évaluation de performance inclut la gestion par objectif avec la fixation d'objectifs mutuels/communs? (performance4) |  |
| Mes évaluations de performance incluent des commentaires sur mon développement? (performance5) |  |
| Les évaluations de performance sont utilisées pour planifier le développement de mes compétences et mes besoins de formations futures? (performance6) |  |

*Note a*: The French version was validated in this study.

*Note b*: Une échelle en sept points avec des réponses à chaque item allant de 1 à 7 a été utilisée : 1 (*Pas du tout en accord*) ; 2 (*Très peu en accord*); 3 (*Un peu en accord*); 4 (*Moyennement en accord*) ; 5 (*Assez en accord*); 6 (*Fortement en accord*); 7 (*Très fortement en accord*).

*List of the 66 initial items and dimensions of the “High Well-Being and Performance Work System Scale, HWBPWS” in French*

| Items | Dimensions de Guest (2017) |
| --- | --- |
| Les processus de recrutement et de sélection de cette organisation sont impartiaux (justes et équitables)? | **1. Investir dans les employés** |
| Il ne semble pas y avoir de favoritisme dans aucune des décisions de recrutement prises ici? |  |
| Des comités d’embauche sont utilisés lors du processus de recrutement et de sélection dans cette organisation ? |  |
| Cette organisation n'a pas besoin de prêter plus d'attention à la façon dont elle recrute des employés? |  |
| J'ai été formé à divers emplois ou pour diverses compétences (polyvalent au niveau de la formation) et/ou j’exerce régulièrement plusieurs emplois (polyvalent sur les postes/rotation des postes)? |  |
| J'ai reçu une formation intensive/approfondie sur les compétences spécifiques à l'entreprise? (ex : formation spécifique à une tâche ou à l’organisation) |  |
| J'ai reçu une formation intensive/approfondie en compétences génériques (non spécifiques à l’entreprise) (résolution de problèmes, aptitudes à la communication, etc.)? |  |
| Des programmes de formation approfondis me sont offerts? |  |
| Je vais normalement suivre des programmes de formation tous les ans? |  |
| Il existe des programmes de formation officiels pour enseigner aux nouveaux employés les compétences dont ils ont besoin pour effectuer leur travail? |  |
| Des formations me sont proposées afin d’accroître mes possibilités d’avancement/promotion dans cette organisation? |  |
| J'ai un cheminement de carrière clair au sein de l'organisation? |  |
| J'ai très peu d'avenir au sein de cette organisation? (Item renversé) |  |
| Mes aspirations professionnelles au sein de l'organisation sont connues de mes supérieurs immédiats? |  |
| Il y a plus d'un poste potentiel sur lequel je peux être promue? |  |
| J'ai la possibilité de discuter des problèmes liés au travail avec mon supérieur immédiat? |  |
| J'ai l'occasion d'examiner les questions liées au travail avec mon supérieur hiérarchique immédiat? |  |
| Mon travail est simple et assez répétitif? (Item renversé) | **2. Offrir un travail stimulant** |
| J'ai beaucoup d'occasions de décider comment faire mon travail? |  |
| Si un problème survient dans mon travail, je peux prendre des mesures pour y remédier? |  |
| J'ai peu d'occasions d'utiliser mon propre jugement lorsque je fais mon travail? (Item renversé) |  |
| Je me sens souvent ennuyé au travail? (Item renversé) |  |
| J'ai assez d'informations pour bien faire mon travail? |  |
| Les informations sur la performance financière de mon organisation sont partagées avec moi? |  |
| L'entreprise informe ses employés de ses performances? |  |
| Je me sens à l'aise de communiquer à la direction des informations qui ne correspondent pas nécessairement à ce qu'elle veut entendre? |  |
| Il m'est facile de communiquer mes pensées à la direction? |  |
| On me donne suffisamment d'informations pour comprendre mon rôle dans cette organisation? |  |
| On me fournit des informations stratégiques pertinentes (par exemple, la mission stratégique, les objectifs d’affaires, des informations sur les concurrents, etc.)? |  |
| J'ai du travail dans mon organisation pour aussi longtemps que je le veux? |  |
| Si je perdais mon poste actuel, mon organisation s'efforcerait de me placer dans une autre fonction ailleurs dans l'organisation? |  |
| Je peux être certain(e) d'être employé(e) dans mon organisation tant et aussi longtemps que je fais du bon travail? |  |
| Cette organisation me fournit une sécurité pour la retraite (fonds de pension)? |  |
| Je ne suis pas vraiment certain(e) de savoir combien de temps je serai à l’emploi de mon organisation? (Item renversé) |  |
| Mes conditions de travail sont bonnes? | **3. Offrir un environnement de travail social et physique positif** |
| Ma santé ne s’est pas détériorée du fait de travailler pour cette organisation? |  |
| Je me sens toujours en sécurité en travaillant ici dans ces conditions? |  |
| Cette organisation fait tout ce qu'elle peut pour assurer le bien-être de ses employés? (e.g., comité santé/mieux-être) |  |
| Cette organisation dépense suffisamment d'argent pour des questions liées à la santé et à la sécurité? |  |
| Je pense que l’organisation consacre suffisamment de ressources à la sensibilisation à l’égalité des chances dans l’accès à l’emploi et à la formation liée à l’accès à l’emploi? |  |
| Je pense que cette organisation aide les employés à trouver un équilibre entre leurs responsabilités professionnelles et familiales/personnelles? |  |
| Je pense que la direction soutient la différence culturelle dans cette organisation? |  |
| Je pense que les hommes et les femmes ont les mêmes opportunités d’emploi dans cette organisation? |  |
| Je pense que l'égalité des chances dans l’accès à l'emploi est facilitée dans cette organisation? |  |
| Mon salaire est entièrement basé sur mon ancienneté au sein de l’entreprise? |  |
| Une partie de ma rémunération dépend de la situation financière de l’organisation? (Item renversé) |  |
| Notre salaire dans cette entreprise est supérieur à ce que proposent nos concurrents? |  |
| Une partie de ma rémunération est basée sur les performances de mon équipe de travail ou de mon département? (Item renversé) |  |
| Je crois que je serais payé plus équitablement si je travaillais dans une autre organisation? (Item renversé) |  |
| Je participe à des programmes conçus pour obtenir la participation et la contribution des employés (par exemple, un cercle de qualité, des équipes de résolution de problèmes ou autres groupes similaires? | **4. Encourager la participation des employés** |
| Les employés doivent régulièrement remplir des sondages afin d’identifier et de corriger des problèmes d’ordre moral? |  |
| J'ai accès à une procédure formelle de résolution de grief/plainte? |  |
| Nous sommes organisés en équipes de travail autogérées pour assumer une grande partie de nos tâches? |  |
| J'ai la possibilité de travailler chez moi ou de la maison pendant les heures de travail normales? | **5. Offrir un soutien organisationnel** |
| Cette organisation autorise les horaires flexibles (c’est-à-dire pas d’heure de début/de fin, mais un nombre défini d’heures de travail par semaine/mois)? |  |
| Cette organisation permet le partage d’emploi (partage d’un emploi à temps plein avec un autre employé)? |  |
| J'ai la capacité de réduire mes heures de travail (par exemple, passer d'un emploi à temps plein à un emploi à temps partiel)? |  |
| Cette organisation permet de comprimer ses heures de travail (c.-à-d. des heures standards travaillées sur moins de jours). |  |
| J'ai la possibilité de changer les heures de travail définies (y compris l’horaire de travail)? |  |
| J'ai la possibilité de travailler uniquement pendant les périodes scolaires? |  |
| Je reçois régulièrement des évaluations formelles de performance? |  |
| Je reçois de la rétroaction formelle provenant de plusieurs sources (des superviseurs, des collègues, etc.)? |  |
| Mon évaluation de performance est basée sur des résultats objectifs et quantifiables?) |  |
| Mon évaluation de performance inclut la gestion par objectif avec la fixation d'objectifs mutuels/communs? |  |
| Mes évaluations de performance incluent des commentaires sur mon développement? |  |
| Les évaluations de performance sont utilisées pour planifier le développement de mes compétences et mes besoins de formations futures? |  |
| *Note :* Une échelle en sept points avec des réponses à chaque item allant de 1 à 7 a été utilisée : 1 (Pas du tout en accord) ; 2 (Très peu en accord); 3 (Un peu en accord); 4 (Moyennement en accord) ; 5 (Assez en accord); 6 (Fortement en accord); 7 (Très fortement en accord). | |

*List of the 66 initial items and dimensions of the “High Well-Being and Performance Work System Scale, HWBPWS” in English*

| Items | Dimensions de Guest (2017) |
| --- | --- |
| The recruitment and selection processes in this organization are impartial (fair and equitable)? | **1. Investing in employees** |
| Favoritism is not evident in any of the recruitment decisions made here? |  |
| Interview panels are used during the recruitment and selection process in this organization? |  |
| This organisation does not need to pay more attention to the way it recruits people? |  |
| I have been trained in a variety of jobs or skills (“cross-trained”) and/or routinely perform more than one job (cross-utilized”)? |  |
| I have received intensive/extensive training in company-specific skills (e.g., task or firm-specific training)? |  |
| I have received intensive/extensive training in generic skills (e.g., problem-solving, communication skills, etc.)? |  |
| Extensive training programs are provided for me? |  |
| I will normally go through training programs every year? |  |
| There are formal training programs to teach new hires the skills they need to perform their jobs? |  |
| Formal training programs are offered to me in order to increase my promotability in this organization? |  |
| I have a clear career path within the organization? |  |
| I have very little future within this organization? (Reverse coded) |  |
| My career aspirations within the company are known by my immediate supervisors? |  |
| I have more than one potential position to be promoted? |  |
| I have the opportunity to discuss work-related problems with my immediate manager? |  |
| I have the opportunity to examine work-related issues with my immediate manager? |  |
| My job is simple and quite repetitive? (Reverse coded) | **2. Engaging work (i.e., providing stimulating work)** |
| I have lots of opportunity to decide how to do my work? |  |
| If a problem emerges with my work, I can take action to remedy it? |  |
| I have little opportunity to use my own judgement when doing my work. (Reverse coded) |  |
| I often feel bored at work? (Reverse coded) |  |
| I have enough information to do my job well? |  |
| Information about how well my organization is doing financially is shared with me? |  |
| The company does let its employees know how it is performing? |  |
| I feel comfortable communicating information to management that is not necessarily what they want to hear? |  |
| It is easy for me to communicate my thoughts to management? |  |
| I am given enough information to understand my role in this organization? |  |
| I am provided with relevant strategic information (e.g., strategic mission, goals, tactics, competitor information, etc.)? |  |
| I have work in my organization for as long as I want it? |  |
| If I were to lose my current position, my organization would try very hard to place me in another position elsewhere in the organization? |  |
| I can be sure of being employed in my organization as long as I do good work? |  |
| This organization provides me with retirement security? |  |
| I am not really sure how long I will be employed by my organization? (Reverse coded) |  |
| My working conditions here are good? | **3. A positive social and physical work environment** |
| My health has not suffered as a result of working for this organisation? |  |
| I always feel safe working here in these conditions? |  |
| This organisation does what it can to ensure the well-being of its employees? (e.g., health / wellness committee) |  |
| This organisation spends enough money on health and safety-related matters? |  |
| I feel that the organisation spends enough resources on equal employment opportunity awareness and employment opportunity-related training? |  |
| I feel that this organization supports employees with the balancing of work and family responsibilities? |  |
| I feel that management is supportive of cultural difference in this organisation? |  |
| I feel that men and women have the same employment opportunities in this organization? |  |
| I feel that equal employment opportunity is promoted in this organization? |  |
| How much I get paid is based totally on how long I have been with the company? |  |
| Part of my compensation is based on how well the organization is doing financially? (Reverse coded) |  |
| Our pay in this company is higher than what competitors offer? |  |
| Part of my compensation is based on how well my workgroup or department performs? (Reverse coded) |  |
| I believe that I would be paid more fairly if I worked at another organization? (Reverse coded) |  |
| I am involved in programs designed to elicit participation and employee input (e.g., quality circle, problem-solving or similar groups? | **4. Voice (i.e., encouraging employee participation)** |
| Employees are routinely administered attitude surveys to identify and correct employee morale problems? |  |
| I have access to a formal grievance/complain resolution procedure? |  |
| We are organized in self-directed work teams in performing a major part of our roles? |  |
| I have the opportunity to work at or from home during normal working hours? | **5. Organizational support** |
| This organization allows flex-time (i.e., no set start/finish time but a set number of hours per week/month to work)? |  |
| This organization allows job-sharing schemes (sharing a full-time job with another employee)? |  |
| I have the ability to reduce working hours (e.g., switching from full-to part-time employment)? |  |
| This organization allows compressed hours (i.e., working standard hours across fewer days)? |  |
| I have the ability to change set working hours (including shift pattern)? |  |
| I have the possibility to work only during school term times? |  |
| I receive formal performance appraisals or evaluation on a routine basis? |  |
| I receive formal performance feedback from more than one source (i.e., feedback from several individuals such as supervisors, peers, etc.) |  |
| My performance appraisal is based on objective, quantifiable results? |  |
| My performance appraisal include management by objective with mutual goal setting? |  |
| My performance appraisals include developmental feedback? |  |
| Performance appraisals are used to plan skill development and training for future advancements? |  |
| *Note :* A seven-point scale with responses to each item ranging from 1 to 7 was used: 1 (Strongly disagree) ; 2 (Disagree); 3 (Somewhat disagree); 4 (Neither agree nor disagree) ; 5 (Somewhat agree); 6 (Agree); 7 (Strongly agree). | |
